# Layers of Rarity: An Unusual Concurrence of Keratoconus, Salzmann’s Nodular Degeneration, Ptosis, and Congenital Retinal Macrovessel

**DOI:** 10.7759/cureus.92115

**Published:** 2025-09-12

**Authors:** Deepsekhar Das, Neiwete Lomi, Aiswarya Sasi, Neha Kumari, Manu Muraleekrishna, Radhika Tandon

**Affiliations:** 1 Ophthalmology, All India Institute of Medical Sciences, New Delhi, IND

**Keywords:** congenital ptosis, congenital retinal macrovessel, keratoconus, optic disc hypoplasia, salzmann nodular degeneration

## Abstract

We report a highly unusual case involving the coexistence of four rare ophthalmic conditions (keratoconus, Salzmann nodular degeneration (SND), congenital ptosis, and congenital retinal macrovessel) in a single patient. A young woman in her late adolescence presented with bilateral progressive vision loss and a history of left upper eyelid drooping since childhood. She was recently diagnosed as being in the early stages of pregnancy. Ocular examination revealed signs consistent with keratoconus, including corneal thinning, conical protrusion, and a scissoring reflex on retinoscopy. Two subepithelial, bluish-gray nodules characteristic of SND were found in the right eye. The patient also demonstrated left-sided congenital ptosis with good levator function and Bell’s phenomenon. Fundus evaluation revealed bilateral optic disc hypoplasia and a congenital retinal macrovessel traversing the fovea in the right eye without associated hemorrhage or cysts.

This case highlights the importance of comprehensive ophthalmologic evaluation when encountering atypical presentations. The association between keratoconus and other systemic or ocular disorders has been well-documented; however, simultaneous manifestation with Salzmann nodular degeneration, congenital ptosis, and congenital retinal macrovessel expands the spectrum of possible syndromic presentations.

## Introduction

Keratoconus is a progressive, bilateral, non-inflammatory ectatic disorder of the cornea characterized by stromal thinning, conical protrusion, and irregular astigmatism that leads to decreased visual acuity [1]. It typically presents during adolescence or early adulthood and may progress over time, often stabilizing by the fourth decade of life [2]. Though the exact etiology remains elusive, several risk factors and associations have been proposed, including genetic predisposition, mechanical eye rubbing, and coexisting atopic or systemic disorders such as Down syndrome, Turner syndrome, and connective tissue disorders like Ehlers-Danlos and Marfan syndrome [3,4]. Histopathologically, keratoconus involves disruptions in the epithelial basement membrane, Bowman’s layer fragmentation, and stromal thinning.

Salzmann nodular degeneration (SND) is a rare, slowly progressive degenerative condition of the cornea characterized by bluish-gray subepithelial nodules composed of hyaline-like material, often located in the mid-peripheral cornea [5]. While commonly asymptomatic, SND can occasionally cause visual impairment due to surface irregularity or central involvement. It typically occurs in eyes with a prior history of chronic ocular surface disease, trauma, or inflammation, but its pathogenesis remains poorly understood [6].

Congenital ptosis refers to drooping of the upper eyelid present at birth, usually due to dystrophic or fibrotic changes in the levator palpebrae superioris muscle. While isolated ptosis is common, its association with corneal ectasias like keratoconus is exceedingly rare. However, mechanical ptosis has been hypothesized to influence corneal biomechanics and contribute to ectatic changes [7].

Congenital retinal macrovessels (CRMs) are rare vascular anomalies wherein an aberrant large retinal vessel (arterial, venous, or both) crosses the macular region. First described in 1869, CRMs are typically incidental findings, but in rare cases can be associated with hemorrhages or macular cysts affecting visual function [8].

While these conditions are individually rare, their simultaneous presentation in a single patient is extraordinary. The literature scarcely documents cases with more than one of these findings in conjunction. A previously reported case associated SND with posterior keratoconus, while another documented keratoconus developing post-ptosis surgery [9,10]. However, no known reports describe the co-occurrence of all four entities (keratoconus, SND, congenital ptosis, and CRMs) in a single patient.

This case report aims to present an unusual confluence of rare ocular conditions in a young female patient. The report not only adds to the existing knowledge of these disorders but also explores potential associations and highlights the challenges in clinical decision-making and management, particularly during pregnancy when therapeutic interventions such as corneal collagen cross-linking must be carefully timed.

## Case presentation

A 26-year-old woman presented to the outpatient ophthalmology department with complaints of gradual, painless diminution of vision in both eyes for the past two years. The visual loss was progressive and associated with intermittent mild ocular itching. The patient also reported drooping of the left upper eyelid since early childhood. There was no history of ocular trauma, prior surgery, or systemic illness. She had recently been diagnosed as pregnant and was in her first trimester.

**Figure 1:**
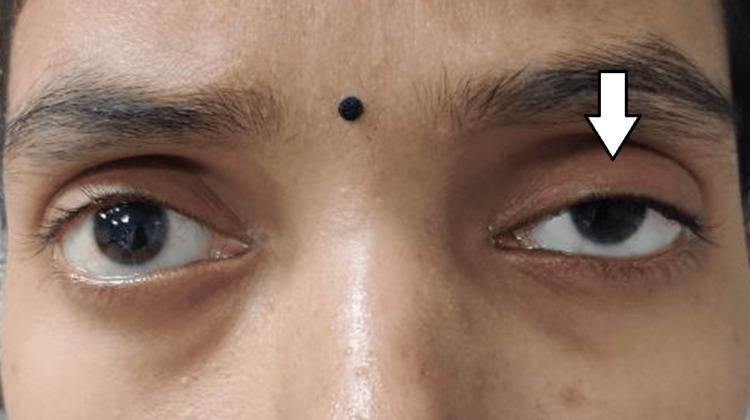
Clinical picture of the patient showing moderate ptosis of the left eye (white arrow), which is evident when compared to the right eye. Note: Written informed consent to include this image in an open-access article was obtained from the patient.

On general physical examination, she appeared healthy with stable vital signs. Her facial features were notable for a flat nasal bridge and midfacial hypoplasia. Ocular examination revealed a best-corrected visual acuity (BCVA) of 6/12 in the right eye and 6/18 in the left eye using a Snellen’s chart. Intraocular pressures were 16 mm Hg bilaterally. A final diagnosis of bilateral keratoconus (advanced in the right eye), SND in the right eye with CRM, and left-sided congenital ptosis (Figure 1) was established. Due to her pregnancy, the patient was not scheduled for immediate corneal collagen cross-linking and was advised to undergo close follow-up for serial monitoring and postpartum treatment planning.

**Figure 2:**
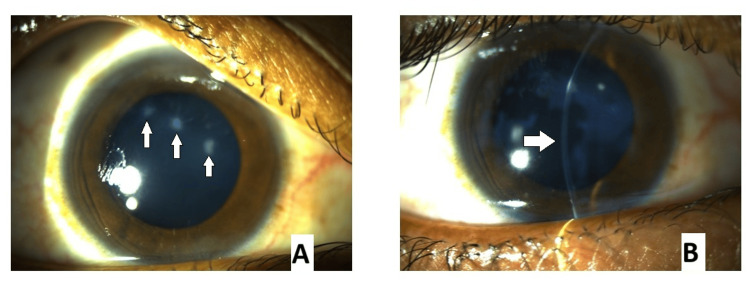
Clinical picture of the right cornea showing (A) bluish-gray nodules, typical of SND (arrows), and (B) the subepithelial location of the lesion, along with thinning in the inferior cornea (arrow)

Eyelid evaluation revealed moderate congenital ptosis of the left upper lid with good Bell’s phenomenon and a levator palpebrae superioris excursion of 10 mm, indicating preserved muscle function. On retinoscopy, a scissoring reflex was noted in both eyes, suggestive of irregular astigmatism. Slit-lamp biomicroscopy revealed mild meibomian gland dysfunction bilaterally. Notably, two distinct, elevated, bluish-gray subepithelial nodules approximately 2 mm in diameter were observed in the right eye, consistent with SND (Figure 2).

The cornea in both eyes exhibited localized thinning and paracentral protrusion. On downgaze, a positive Munson sign confirmed the clinical suspicion of keratoconus. The remainder of the anterior segment examination was unremarkable.

**Figure 3:**
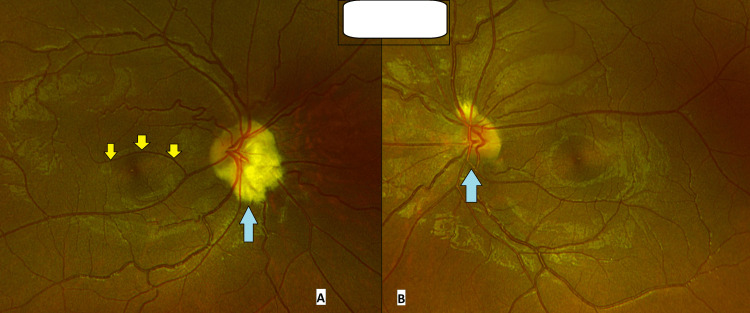
Fundus picture of the (A) right and (B) left eye showing eye optic disc hypoplasia (marked with blue arrows), along with a congenital retinal macrovessel (yellow arrows) in the right eye

Fundus examination disclosed bilateral hypoplastic optic discs. In the right eye, an anomalous large retinal vessel coursed across the fovea, consistent with a CRM. There were no associated hemorrhages or cystoid changes on clinical examination (Figure 3).

**Figure 4:**
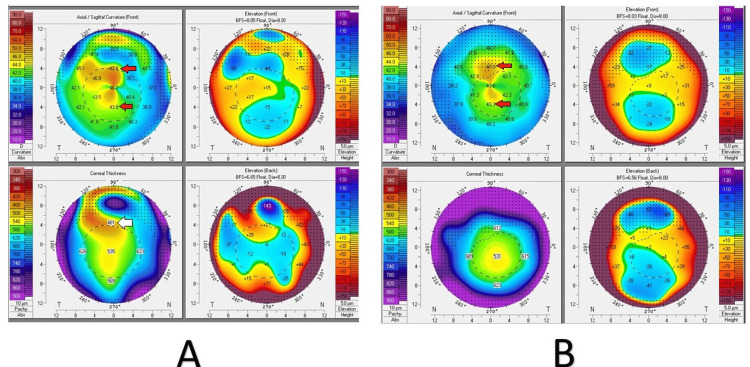
Clinical picture of Pentacam map of both eyes (A) Pentacam images of the right cornea showing severe astigmatism (red arrows) with paracentral corneal thinning (white arrow). (B) Pentacam images of the left cornea showing astigmatism (red arrows) with inferior corneal thinning.

**Figure 5:**
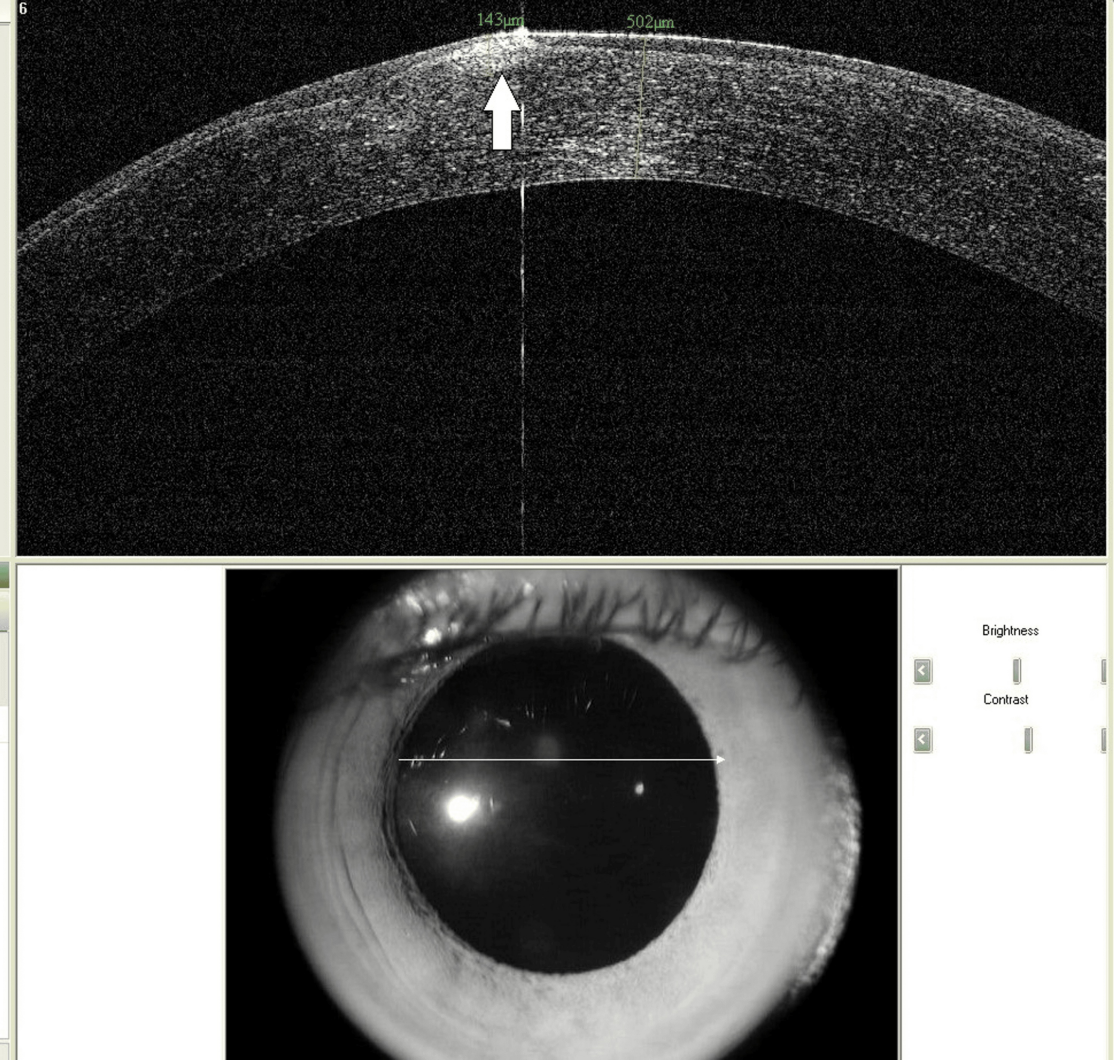
Anterior segment optical coherence tomography (OCT) of the right eye showing subepithelial location of the nodule (white arrow)

To further characterize the corneal condition, a Pentacam corneal tomography was performed. It showed significant astigmatism of 6.5 diopters in the right eye and 5.3 diopters in the left eye. Kmax values were 48.6 D in the right eye and 46.7 D in the left eye. The thinnest corneal pachymetry measured 438 µm and 527 µm in the right and left eye, respectively. Anterior segment optical coherence tomography (OCT) confirmed the presence of subepithelial corneal nodules in the right eye (Figure 4, Figure 5).

## Discussion

The current case presents an unprecedented cluster of four distinct ocular pathologies (keratoconus, SND, congenital ptosis, and CRM) coexisting in a single patient. Each of these conditions is relatively uncommon in clinical practice, and their synchronous manifestation raises questions regarding possible underlying genetic, structural, or embryologic associations.

Keratoconus, typically presenting in the second decade of life, is a progressive, bilateral ectasia of the cornea. It leads to high myopic astigmatism and corneal scarring in advanced cases, often resulting in significant visual disability [1,2]. The pathogenesis of keratoconus is multifactorial, with genetic, biochemical, mechanical (e.g., eye rubbing), and hormonal factors being implicated. Its association with systemic syndromes such as Down syndrome, Turner syndrome, and connective tissue disorders is well established [3,4]. Notably, hormonal changes in pregnancy may accelerate keratoconus progression due to increased corneal laxity; hence, caution is needed when considering interventions like collagen cross-linking in pregnant patients [5].

SND is a rare, non-inflammatory corneal degeneration involving the subepithelial region, typically occurring secondary to chronic ocular surface disorders [6]. The nodules are composed of hyaline and fibrous tissues and are most often asymptomatic unless centrally located. While the coexistence of SND and keratoconus is rare, they share potential triggers such as ocular surface inflammation and mechanical irritation [7]. Literature reveals only one case where SND was reported in a patient with posterior keratoconus [8].

Congenital ptosis results from a developmental myogenic dysgenesis of the levator muscle. While commonly isolated, some studies have hypothesized that ptosis can indirectly influence corneal shape by altering eyelid pressure dynamics [9]. There is limited but intriguing evidence suggesting that either congenital or postoperative changes in lid mechanics could contribute to ectatic corneal changes [10]. In this case, the congenital ptosis did not appear to be causative but might have acted as a modifying factor in the disease presentation.

CRM is an extremely rare vascular anomaly involving large aberrant vessels traversing the fovea. Most CRMs are venous and do not affect vision, though complications such as hemorrhages and foveal cysts have been documented [11]. Our patient had a macrovessel in the right eye without visual sequelae. Interestingly, no prior reports link CRM with either keratoconus or SND, making this association novel.

The combination of these entities in one individual does not fit into any known ocular syndrome. While the coexistence could theoretically be coincidental, it raises the possibility of an unrecognized syndromic or developmental basis. One speculative theory involves disruptions in neural crest-derived tissues, which contribute to both anterior segment structures and retinal vasculature. Alternatively, this case may represent the phenotypic variability of an undiagnosed genetic condition.

The management of such a complex case must be cautious, particularly in the context of pregnancy. Corneal collagen cross-linking is generally deferred during gestation due to the influence of hormonal changes on corneal biomechanics and the need to avoid procedural stress or potential teratogenic risk from agents like riboflavin [12]. Conservative visual rehabilitation and close monitoring were deemed appropriate in this instance.

This case underscores the need for a multidisciplinary approach to rare ocular presentations, combining corneal, retinal, and neuro-ophthalmologic expertise. Genetic counseling and systemic evaluation might also be prudent in similar presentations.

## Conclusions

This case highlights a remarkably rare concurrence of four distinct ocular anomalies in a single patient: keratoconus, SND, congenital ptosis, and CRM. Each of these conditions individually presents unique diagnostic and management challenges, and their simultaneous occurrence has not been previously reported in the literature. This case underscores the importance of a thorough clinical evaluation and multimodal imaging in patients presenting with atypical visual complaints, especially in young individuals.

The overlapping of anterior segment and posterior segment abnormalities, along with a congenital eyelid malposition, raises the possibility of a broader developmental or syndromic association. While this could be coincidental, it warrants further genetic and embryologic exploration. Given the patient’s pregnancy, conservative management was prioritized, with a plan for definitive interventions post-partum. This case emphasizes the need for personalized, multidisciplinary care in patients with complex ocular presentations to optimize visual outcomes and long-term management.
